# Tumor sidedness is not an independent prognostic marker of colorectal cancer patients undergoing curative resection: A retrospective cohort study

**DOI:** 10.1371/journal.pone.0218207

**Published:** 2019-06-12

**Authors:** Joseph Chung Yan Chan, Connie Irene Diakos, Alexander Engel, David Lok Hang Chan, Nick Pavlakis, Anthony Gill, Stephen John Clarke

**Affiliations:** 1 Bill Walsh Translational Research Laboratories, Kolling Institute of Medical Research, St Leonards, New South Wales, Australia; 2 Sydney Medical School, University of Sydney, Sydney, New South Wales, Australia; 3 Northern Sydney Cancer Center, Royal North Shore Hospital, St Leonards, New South Wales, Australia; 4 Department of Colorectal Surgery, Royal North Shore Hospital, St Leonards, New South Wales, Australia; 5 Cancer Diagnosis and Pathology Group, Kolling Institute of Medical Research, St Leonards, New South Wales, Australia; VA Boston Healthcare System, Harvard Medical School (Brigham and Women’s Hospital), UNITED STATES

## Abstract

**Background:**

Recent literature has suggested that tumor sidedness in colorectal cancer (CRC) is an independent prognostic and potentially predictive marker of survival. The aims of the study were to determine the prognostic significance of tumor sidedness in colorectal cancer patients undergoing primary tumor resection and to assess associated tumor biology.

**Methods:**

A total of 3281 consecutive patients who underwent surgical resection of their primary CRC from January 1998 to December 2012 were analyzed for association with tumor biologic factors and with overall survival. Metastatic patients were excluded from analysis.

**Results:**

Left sided CRCs were associated with a number of additional key prognostic markers including BRAFV600E wildtype status (P<0.001), mismatch repair proficiency (p<0.001), absence of peritumoral lymphocytic response (p = 0.001), high lymphocyte-to-monocyte ratio (p<0.001) and low neutrophil-to-lymphocyte ratio (p<0.001). In primary analysis with 3067 patients, there was no statistical difference in sidedness in the univariate analysis (p = 0.291). Three further subgroup analyses were performed. In the first subgroup, only stage III patients were analyzed. In the second, patients with mismatch repair deficiency were removed. In the third, additional clinicopathologic variables known to be independently prognostic were added into analysis. In all three subgroup analyses tumor sidedness was not an independent prognostic marker.

**Conclusions:**

Tumor sidedness was not an independent prognostic marker of CRC. However, right sided CRCs were associated with several key independent prognostic markers supporting a hypothesis that tumor sidedness is a surrogate for other biomarkers.

## Introduction

Colorectal cancer (CRC) is one of the most commonly diagnosed cancers worldwide with data from the World Health Organization estimating that up to 1.4 million people are diagnosed annually [1]. Survival rates from CRC in the Western world have improved particularly in the metastatic setting where targeted therapies, combined with advances in molecular testing, have been introduced [2]. However, significant gaps in our knowledge of CRC and the lack of effective biomarkers for treatment and prognostic stratification remain problematic, especially in the post-operative adjuvant setting.

Current knowledge suggests that prognosis is dependent on numerous clinicopathological variables including demographic factors, mutational status and inflammatory markers [3]. Recently, one area that has gained prominence has been the anatomic site of the primary cancer [4]. Research into this area was initially driven by an observed increase in right-sided CRC in retrospective analysis of the SEER dataset [5, 6]. Since then, several studies investigating anatomic site have found that there may be survival differences based on the sidedness of the primary tumor [4]. Despite growing evidence, there is ongoing debate as to whether tumor sidedness alone is an independent prognostic marker. Some clarity has been provided by the latest and most complete meta-analysis that suggested left-sided cancer carries a better prognosis [4]. Contextually, these findings have gained further traction and importance because of re-examinations of clinical trial data in metastatic CRC patients [7]. In several studies, sidedness was found not only to be prognostic in favor of left-sided cancer but also predictive of treatment outcome, with improved survival associated with use of combination chemotherapy plus epidermal growth factor receptor (EGFR) targeted antibodies [7, 8].

A number of plausible hypotheses have been proposed to explain these observed differences in survival based on sidedness. These include differences in embryological origin and localization of gut flora [9]. More recently, there has been further evidence that colorectal cancer is comprised of multiple distinct molecular subgroups. Guinney *et al*. (2015) separated colorectal cancer into four distinct molecular subtypes with certain subtypes more likely to be enriched to either the right or left sides of the colon [10].

The molecular subtypes of CRC give credibility to the hypothesis that sidedness is a surrogate marker for clinicopathologic factors. Prior studies with large patient cohorts have cited the lack of key pathological variables such as microsatellite instability as a central limitation. Moreover, datasets like SEER often lack additional markers which have previously been shown to be prognostic.

In prior large studies, the lack of key clinicopathologic data such as microsatellite instability data has been cited as a significant limitation [6]. These clinicopathological factors may also vary significantly between stages and explain survival differences in sidedness that have been observed across tumor stages [11]. Performing specific analyses on patients with genomic differences and also across stages may provide a better understanding of survival variations observed based on tumor sidedness. In addition, we have noted that large datasets such as SEER also often lack additional clinicopathological markers known previously to be highly prognostic. We hypothesize that sidedness is a surrogate of these markers.

The current study attempts to address these shortcomings of the prior literature regarding sidedness and survival in the setting of colorectal cancer patients undergoing curative resection. Specifically, we aimed to address the lack of inclusion of additional key confounders such as mutation status and inflammatory markers. The primary goal was to re-examine sidedness as a prognostic marker and to clarify this by including additional clinicopathological variables previously demonstrated to be independently prognostic. Our secondary goal was to investigate whether factors of tumor biology, including mismatch repair (MMR) status, BRAFV600E mutant status and inflammatory markers are associated with tumor sidedness and whether this can support an alternate hypothesis for these observed survival differences.

## Materials and methods

### Patient cohort

A retrospective analysis of a prospectively collected clinicopathological data base was conducted. Patient data were collected for 3281 consecutive patients at the Northern Sydney Local Health District (NSLHD, Sydney, Australia) who had undergone primary resection of colorectal cancer between January 1998 and December 2012. Data were drawn from six hospitals, including two major quaternary and four community hospitals, all of which had general or specialist colorectal surgical units. Gender, age and histologic grading were prospectively collected. Patient tumour samples were all re-reviewed and staged according to the AJCC 7^th^ edition 2009 staging system [12]. Tumor sidedness was defined as follows, right sided CRC was from caecum to the splenic flexure including the transverse colon. Left sided CRC represented the remaining large bowel including the rectum.

The treatment patients received differed depending on stage but were all within accepted national guidelines. The majority of patients with high risk stage II and III colon cancer were offered standard adjuvant chemotherapy and most patients with stage II and III rectal cancer were offered neoadjuvant chemotherapy. Treatments were delivered at multiple centres including both public and private oncology treatment centres.

Patients were followed up every 3–6 months in most cases. This consisted of a full clinical history, physical examination and blood tests including full blood count, plasma biochemistry, serum levels of carcinoembryonic antigen (CEA) and a single computed tomography (CT) of chest, abdomen and pelvis 12–18 months postoperatively [13].

### Immunohistochemistry

Resected tumour specimens were evaluated for BRAFV600E mutation and mismatch repair (MMR) status using immunohistochemistry methods that we have demonstrated to be highly sensitive and specific [14, 15].

### Survival data

The primary endpoint for analysis was overall survival (OS), measured from the date of surgery until date of last follow up or date of death from any cause. Follow up data were collected from a variety of sources including hospital pathology databases, private offices, electronic medical records, central death registries and publicly available death records up to November 2015. Median follow up was 53 months (interquartile range 26–90 months) with 1070 deaths from any cause.

### Statistics

The lymphocyte-to-monocyte ratio (LMR) and neutrophil-to-lymphocyte ratio (NLR) were constructed by taking the ratio of the absolute count of the respective component of the full blood count. Patient data were divided into ‘low’ and ‘high’ groups using a cutpoint of 2.86 and 3.75 for LMR and NLR respectively. These cutpoints were derived from the R package Maxstat as previously described [16]. The lymph node ratio (LNR) was calculated by dividing the total number of positive nodes over the total number of nodes sampled. Patients were separated into ‘low’ and ‘high’ groups using a cutpoint of 0.31 using the aforementioned method.

Associations between sidedness and other clinicopathologic variables were performed using chi squared tests. Univariate analyses and multivariate analyses were performed using a backwards conditional Cox-regression model.

For all statistical tests a p value of < 0.05 was considered significant. Analyses were performed using SPSS software version 20 (SPSS Inc., Chicago, IL, USA) and R (Version 3.2.5). This study was approved by the NSLHD Human Research Ethics Committee under protocol 1201-035M and RESP/14/97.

## Results

### Baseline characteristics

Data from 3281 consecutive patients at the North Sydney Local Health District (NSLHD) who underwent surgical resection of their colorectal adenocarcinoma from January 1998 to December 2012 inclusive were included for analysis. Histologies other than adenocarcinoma and patients with metastatic disease were excluded. Baseline characteristics of patients in the current cohort can be seen in Table 1. The majority of patients in the cohort were older than 70 years (57.3%) and half were male (50.1%). More patients had left-sided (54.2%) than right-sided colon cancer (45.8%). The majority of patients T stage 3 (52.7%) or 4 (22.9%) disease early N stage 0 (56.0%). The full clinicopathologic variable breakdown can be seen in Table 1. Median follow up was 53 months (interquartile range 26–90 months).

**Table 1:** Baseline characteristics of the cohort and the association of clinicopathologic variables with colorectal cancer sidedness.

Clinicopathologic variables	Total (No., %)	Right-sided CRC	Left-sided CRC	*P*
**Age (n = 3070)**				
≤70	1312 (42.7%)	469	843	<0.001
>70	1758 (57.3%)	936	822	
**Gender (n = 3070)**				
Male	1537 (50.1%)	609	928	<0.001
Female	1533 (49.9%)	796	737	
**T stage (n = 3070)**				
1	219 (7.1%)	81	138	<0.001
2	530 (17.3%)	179	351	
3	1619 (52.7%)	756	863	
4	702 (22.9%)	389	313	
**N stage (n = 3070)**				
0	1718 (56.0%)	811	907	0.047
1	887 (28.9%)	375	512	
2	465 (15.1%)	219	246	
**Grade (n = 2673)**				
Low	1344 (50.3%)	586	758	<0.001
Mod	824 (30.8%)	337	487	
High	505 (18.9%)	308	197	
**Thin walled vessel invasion (TWVI) (n = 1626)**				
Absent	1045 (64.3%)	469	576	0.051
Present	581 (35.7%)	290	291	
**Discontinuous extratumoral nodules (DETN) (n = 1658)**				
Absent	1346 (81.2%)	636	710	0.243
Present	312 (18.8%)	136	176	
**Peritumoral lymphocytic response (PTLR) (n = 1669)**				
Absent	597 (35.8%)	247	350	0.001
Present	1072 (64.2%)	533	539	
**Extra venous permeation (EVP) (n = 1915)**				
Absent	1366 (81.4%)	652	714	0.103
Present	312 (18.6%)	133	179	
**BRAF V600E status (n = 2640)**				
Wildtype (wt)	2091 (79.2%)	790	1301	<0.001
Mutant (V600E)	549 (20.8%)	426	123	
**Mismatch repair (MMR) status (n = 2650)**				
Proficient (p)	2185 (82.5%)	834	1351	<0.001
Deficient (d)	465 (17.5%)	391	74	
**Lymphocyte-to-monocyte ratio (LMR) (n = 2175)**				
Low (≤2.86)	1109 (51.0%)	551	558	<0.001
High (>2.86)	1066 (4.9.0%)	450	616	
**Neutrophil-to-lymphocyte ratio (NLR) (n = 2175)**				
Low (≤3.75)	1139 (52.4%)	489	650	0.002
High (>3.75)	1036 (47.6%)	512	524	
**Apical node status (n = 2760)**				
Negative	2535 (91.8%)	1174	1361	0.043
Positive	225 (8.2%)	120	105	
**Lymph node ratio (LNR) (n = 3065)**				
Low (≤0.31)	2707 (88.3%)	1248	1459	0.270
High (>0.31)	358 (11.7%)	154	204	
**Tumor Site (n = 3232)**				
Right	1405 (45.8%)	1405	-	NA
Left	1665 (54.2%)	-	1665	

### Cancer sidedness and association with clinicopathological variables

The associations between colorectal cancer sidedness and clinicopathological variables were explored (Table 1). There was a significant association between right-sided colorectal cancer and older age (p<0.001), female gender (p<0.001), apical node status (p = 0.043), presence of peritumoral lymphocytic response (p = 0.001), positive BRAFV600E mutant status (p<0.001), positive MMR status(p<0.001), a low LMR (P<0.001) and a high NLR (p = 0.002). In addition, there was also an association between sidedness and T stage (p<0.001), N stage (p = 0.047), and tumor grade (p<0.001).

### Cancer sidedness and survival

**Fig 1:**
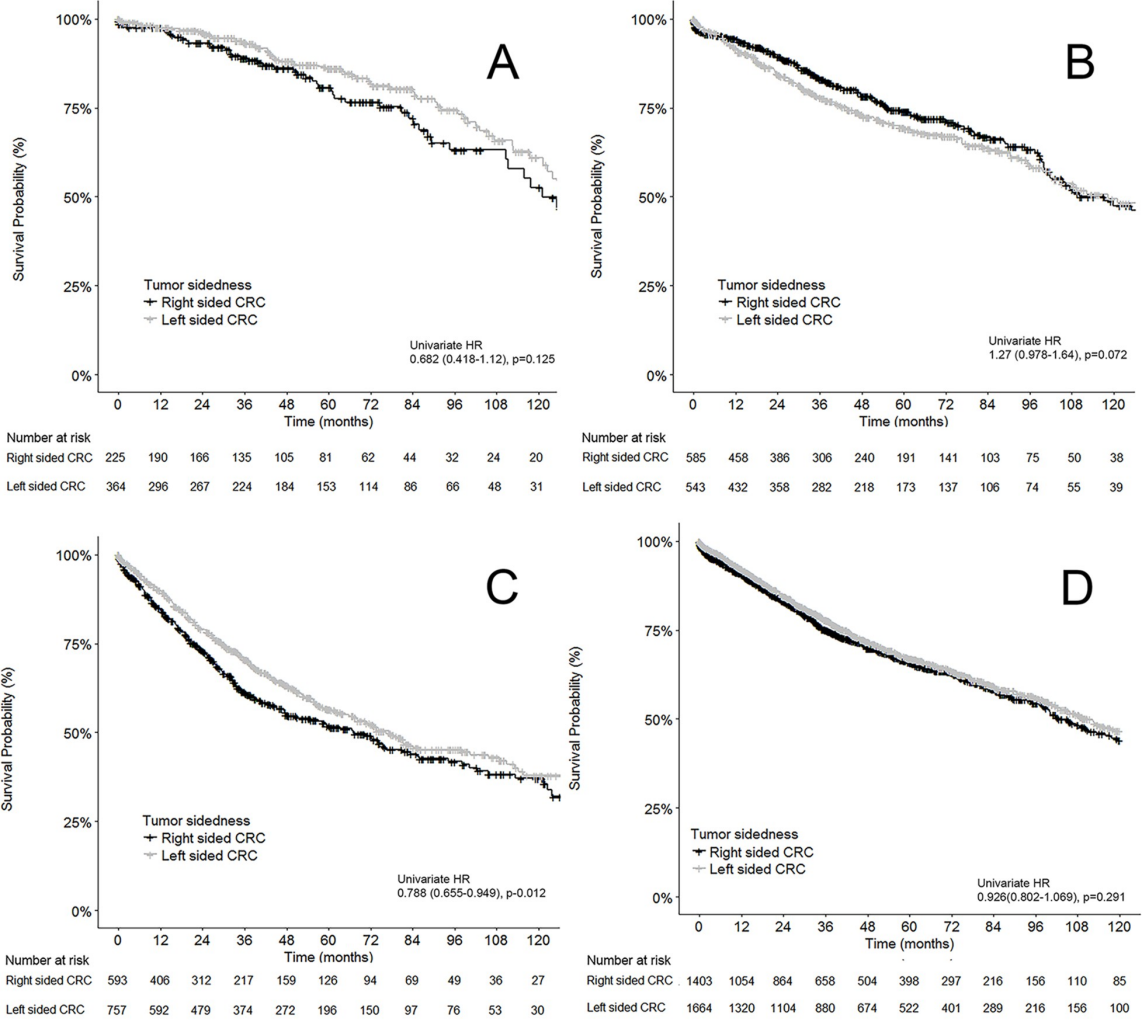
Kaplan Meier curves of the relationship between overall survival and CRC tumor sidedness. A: Stage I CRC, B: Stage II CRC, C: Stage III CRC, D: Combined stage I-III CRC.

The 5-year overall survival (OS) of our total cohort was 66.6%. By site, left-sided CRCs had a 5-year OS of 67.1% compared to right-sided CRC OS of 66.0%. Univariate analysis showed no significant difference between the two sides (p = 0.291) (Fig 1D).

As expected, stage 1 CRC patients had the highest OS with 84.1% alive at 5 years. With respect to sidedness, stage 1 left-sided CRC had a 5yr OS of 86.0% compared to 80.7% 5yr OS in stage 1 right-sided CRC. This difference in survival between tumor sidedness was not statistically significant (p = 0.125) (Fig 1A). For stage 2 patients, left-sided stage 2 CRC had a 69.5% 5yr OS compared to 74.0% in stage 2 right-sided CRC. The survival difference based on sidedness did not reach statistical significance in univariate testing (p = 0.072) (Fig 1B). Patients with stage 3 CRC had a 5yr OS of 54.5%. Stage 3 patients with left sided CRC had a 5yr OS of 56.6%, which was higher than stage 3 patients with right-sided CRC (51.9%). This difference in survival between sides in stage 3 patients was significant in univariate testing (HR 0.788, 95% CI 0.655–0.949, p = 0.012) (Fig 1C).

### Primary multivariate analysis

The relationship between clinicopathological variables and overall survival was assessed initially through a univariate Cox regression model and then through a backwards multivariate Cox-regression model. Variables that were significant in the univariate model were utilized in multivariate analysis (Table 2). In the primary analysis we limited this to include gender, age, T stage, N stage and site. Gender (p = 0.016), Age (p<0.001), T stage(p<0.001) and N stage (p<0.001) were significant but tumor sidedness was not (p = 0.291). In the multivariate analysis gender (p = 0.001), age (p<0.001), T stage (p<0.001) and N stage (p<0.001) all remained significant (Table 2).

**Table 2:** Primary univariate and cox regression multivariate analysis of clinicopathologic variables in relation to overall survival in the combined stage I-III cohort.

Clinicopathologic variables	No., (%), (n = 3067)	Univariate analysis, HR (95% CI)	P	Multivariate analysis, HR (95% CI)	P
**Gender**					
M	1536 (50.1%)	1 (referent)	0.016	1 (referent)	0.001
F	1531 (49.9%)	0.839 (0.727–0.968)_		0.837 (0.727–0.968)	
**Age**					
≤70	1311 (42.7%)	1 (referent)	<0.001	1 (referent)	<0.001
>70	1756 (57.3%)	1.864 (1.599–2.173)		2.289 (1.984–2.640)	
**T stage**					
1	219 (7.1%)	1 (referent)	<0.001	1 (referent)	<0.001
2	529 (17.2%)	0.923 (0.574–1.485)		0.954 (0.648–1.405)	
3	1617 (52.7%)	2.319 (1.535–3.502)		1.575 (1.114–2.228)	
4	702 (22.9%)	5.287 (3.485–8.020)		2.870 (2.003–4.112)	
**N stage**					
0	1717 (56.0%)	1 (referent)	<0.001	1 (referent)	<0.001
1	885 (28.8%)	1.665 (1.405–1.974)		1.369 (1.172–1.598)	
2	465 (15.2%)	3.649 (3.058–4.356)		2.669 (2.234_3.189)	
**Site**					
Right-sided	1403 (45.7%)	1 (referent)	0.291		
Left-sided	1664 (54.3%)	0.984 (0.802–1.069)			

A sensitivity analysis was performed with rectal cancers removed from left sided CRCs. In the repeat analysis, the overall result that sidedness was not significant did not change in univariate analysis (p = 0.884). The other factors remained significant in further multivariate analysis (all p<0.001).

### Subgroup analyses

Three further subgroup analyses were performed. In the first subgroup analysis we analyzed only stage 3 patients (Table 3). In the univariate analysis age, T stage, N stage, grade and combined MMR-BRAF status were all highly significant (all p <0.001). Tumor sidedness was also significant (p = 0.012). In the multivariate analysis all factors remained significant with p value of <0.001 except sidedness (p = 0.716) and combined MMR-BRAF status (p = 0.182) (Table 3).

**Table 3:** Subgroup univariate and cox regression multivariate analysis of clinicopathologic variables in relation to overall survival in stage III patients only.

Clinicopathologic variables	No., (%), (n = 1350)	Univariate analysis, HR (95% CI)	P	Multivariate analysis, HR (95% CI)	P
**Gender**					
M	683 (50.6%)	1 (referent)	0.110		
F	667 (49.4%)	0.860 (0.714–1.035)			
**Age**					
<70	617 (45.7%)	1 (referent)	<0.001	1 (referent)	<0.001
>70	733 (54.3%)	1.862 (1.538–2.254)		1.811 (1.448–2.264)	
**T stage**					
1	32 (2.4%)	1 (referent)	<0.001	1 (referent)	<0.001
2	127 (9.4%)	0.765 (0.252–2.232)		1.048 (0.298–3.679)	
3	715 (53.0%)	2.935 (1.092–7.889)		2.101 (0.669–6.601)	
4	476 (35.3%)	5.467 (2.033–14.703)		4.008 (1.273–12.612)	
**N stage**					
1	885 (65.6%)	1 (referent)	<0.001	1 (referent)	<0.001
2	465 (34.4%)	2.186 (1.816–2.632)		2.058 (1.643–2.579)	
**Site**					
Right-sided	593 (43.9%)	1 (referent)	0.012		0.716
Left-sided	757 (56.1%)	0.788 (0.655–0.949)			
**Grade** *					
Low	495 (42.4%)	1 (referent)	<0.001	1 (referent)	<0.001
Mod	359 (30.7%)	1.276 (1.001–1.626)		1.567 (1.204–2.040)	
High	314 (26.9%)	1.872 (1.468–2.388)		1.705 (1.307–2.225)	
**MMR-BRAF status** *					
MMRp/BRAFV600E	142 (12.0%)	1 (referent)	<0.001		0.182
MMRd/BRAFwt	40 (3.4%)	0.372 (0.185–0.747)			
MMRd/BRAFV600E	120 (10.1%)	0.660 (0.447–0.975)			
MMRp/BRAFwt	882 (74.5%)	0.565 (0.431–0.741)			

*182 patients missing grade, 166 patients missing MMR-BRAF

In the second sub-analysis we re-examined all patients with the inclusion of BRAFV600E mutation status and tumor grade whilst removing all patients with known mismatch repair deficiency. Of the 2156 patients examined, age, T stage, N stage, grade and BRAF status were all significant (p values <0.001) in the univariate analysis. Tumor sidedness was also significant (p = 0.022). In the multivariate analysis, age, T stage, N stage and grade were significant (all pval <0.001). Sidedness again was not significant (p = 0.326) nor was the BRAF status (p = 0.383) (Table 4).

**Table 4:** Subgroup univariate and multivariate analysis of clinicopathologic variables in relation to overall survival in patients without mismatch repair deficiency.

Clinicopathologic variables	No., (%), (n = 2156)	Univariate analysis, HR (95% CI)	P	Multivariate analysis, HR (95% CI)	P
**Gender**					
M	1136 (52.7%)	1 (referent)	0.100		
F	1020 (47.3%)	0.869 (0.736–1.027)			
**Age**					
<70	982 (45.5%)	1 (referent)	<0.001	1 (referent)	<0.001
>70	1174 (54.5%)	1.883 (1.582–2.242)		1.936 (1.602–2.340)	
**T stage**					
1	147 (6.8%)	1 (referent)	<0.001	1 (referent)	<0.001
2	368 (17.1%)	0.674 (0.396–1.145)		0.706 (0.395–1.261)	
3	1113 (51.6%)	1.948 (1.251–3.034)		1.559 (0.948–2.564)	
4	527 (24.4%)	4.099 (2.618–6.419)		2.907 (1.744–4.844)	
**N stage**					
0	1132(52.5%)	1 (referent)	<0.001	1 (referent)	<0.001
1	660 (30.6%)	1.531 (1.256–1.868)		1.305 (1.047–1.626)	
2	364 (16.9%)	3.477 (2.837–4.260)		2.693 (2.110–3.436)	
**Site**					
Right-sided	723 (38.6%)	1 (referent)	0.022		0.326
Left-sided	1148 (61.4%)	0.821 (0.694–0.972)			
Grade*					
Low	921 (49.2%)	1 (referent)	<0.001	1 (referent)	<0.001
Mod	671 (35.9%)	1.357 (1.109–1.660)		1.489 (1.214–1.926)	
High	279 (14.9%)	2.160 (1.691–2.759)		1.629 (1.266–2.095)	
**BRAF**					
Wildtype	1924 (89.2%)	1 (referent)	<0.001	1 (referent)	0.383
Mutant	232 (10.8%)	1.711 (1.354–2.163)		1.161 (0.890–1.513)	

* 285 patients missing grade

In the third sub-analysis we included further clinicopathological variables known to be independently prognostic. Only 779 patients had complete data available for this analysis. The composition of this subgroup was not significantly different to the primary cohort by age, gender, T stage, N stage or tumor site (all p >0.05). In this subgroup, T stage, N stage, Grade, TWVI, DETN, EVP, LMR, NLR, MMR-BRAF status, apical node status and LNR were all significant in the univariate analysis with p values of <0.001. Age (p = 0.002) and PTLR (p = 0.017) were also significant in the univariate analysis. Tumor site was not significant (p = 0.820). In the multivariate analysis, age (p<0.001), T stage (p<0.001), grade (p = 0.012), EVP (p = 0.027), MMR-BRAF status (p = 0.001), LMR (p<0.001) and LNR (p<0.001) remained significant (Table 5).

**Table 5:** Subgroup univariate and multivariate analysis of clinicopathologic variables in relation to overall survival in patients with additional independent prognostic biomarkers.

Clinicopathologic variables	No., (%), (n = 779)	Univariate analysis, HR (95% CI)	P	Multivariate analysis, HR (95% CI)	P
**Gender**					
M	378 (48.5%)	1 (referent)	0.844		
F	401 (51.5%)	0.971 (0.727–1.298)			
**Age**					
≤70	318 (40.8%)	1 (referent)	0.002	1 (referent)	<0.001
>70	461 (59.2%)	1.639 (1.205–2.230)		1.828 (1.325–2.521)	
**T stage**					
1	58 (7.4%)	1 (referent)	<0.001	1 (referent)	<0.001
2	111 (14.2%))	1.065 (0.370–3.066)		0.981 (0.340–2.828)	
3	421 (54.0%)	2.562 (1.041–6.304)		1.903 (0.767–4.719)	
4	189 (24.3%)	7.198 (2.911–17.799)		3.423 (1.347–8.694)	
**N stage**					
0	458 (58.8%)	1 (referent)	<0.001		0.427
1	198 (25.4%)	1.784 (1.262–2.523)			
2	123(15.8%)	3.180 (2.228–4.539)			
**Site**					
Left-sided	372 (47.8%)	1 (referent)	0.820		
Right-sided	405 (52.2%)	1.034 (0.744–1.383)			
**Grade**					
Low	628 (80.6%)	1 (referent)	<0.001	1 (referent)	0.012
High	151 (19.4%)	2.105 (1.532–2.894)		1.638 (1.116–2.404)	
**Thin walled vessel invasion (TWVI)**					
Absent	495 (63.5%)	1 (referent)	<0.001		
Present	284 (36.5%)	2.400 (1.795–3.209)			
**Discontinuous extratumoral nodules (DETN)**					
Absent	629 (80.7%)	1 (referent)	<0.001		
Present	150 (19.3%)	3.274 (2.411–4.445)			
**Peritumoral lymphocytic response (PTLR)**					
Absent	274 (35.2%)	1 (referent)	0.017		
Present	505 (64.8%)	0.699 (0.520–0.938)			
**Extra venous permeation (EVP)**					
Absent	612 (78.6%)	1 (referent)	<0.001	1 (referent)	0.027
Present	167 (21.4%)	3.068 (2.269–4.148)		1.504 (1.049–2.159)	
**MMR-BRAF status**					
MMRp/BRAFV600E	49 (6.3%)	1 (referent)	<0.001	1 (referent)	0.003
MMRd/BRAFwt	53 (6.8%)	0.321 (0.158–0.653)		0.657 (0.317–1.361)	
MMRd/BRAFV600E	101 (13.0%)	0.192 (0.099–0.374)		0.292 (0.148–0.576)	
MMRp/BRAFwt	576 (73.9%)	0.338 (0.213–0.536)		0.768 (0.472–1.252)	
**LMR**					
Low (≤2.86)	376 (48.3%)	1 (referent)	<0.001	1 (referent)	<0.001
High (>2.86)	403 (51.7%)	0.397 (0.293–0.538)		0.531 (0.388–0.727)	
**NLR**					
Low (≤3.75)	433 (55.6%)	1 (referent)	<0.001		
High (>3.75)	346 (44.4%)	2.237 (1.665–3.005)			
**Apical node status**					
Negative	714 (91.7%)	1 (referent)	<0.001		
Positive	65 (8.3%)	4.149 (2.834–6.072)			
**Lymph node ratio**					
Low (≤0.31)	692 (88.8%)	1 (referent)	<0.001	1 (referent)	<0.001
High (>0.31)	87 (11.2%)	4.138 (2.937–5.831)		2.179 (1.447–3.281)	

## Discussion

The current study successfully achieved the aims of investigating the association of primary CRC tumor sidedness with survival and the tumor biology of cancer sidedness. To date, this is one of the largest cohorts with the inclusion of mismatch repair status data to address CRC tumor sidedness.

The principal finding of the current study is that tumor sidedness is not an independent prognostic marker. This result differs from recent literature suggesting survival differences in colorectal cancer (CRC) based on tumor sidedness. Although prior studies had offered conflicting answers, the recent meta-analysis by Petrelli *et al*. (2017) appeared to provide clarity. The study presented pooled multivariate data from 66 studies and demonstrated that left-sided CRC has a small survival benefit with a hazard ratio of 0.82 (95% CI 0.79–0.84, p<0.001) [4]. However, closer examination of the study revealed weaknesses that limit its overall significance. First, there was significant heterogeneity across the included studies. Many studies consisted of specific population types such as the elderly and also missed key prognostic covariates such as microsatellite instability (MSI) status [6, 17]. Secondly, many of the heavily weighted studies included in the analysis drew their data from subsets of the same SEER data, with 11 of the 12 most heavily weighted studies drawn from SEER.[6, 17–19] Interestingly, re-analyses of the SEER data over the years have yielded differing results. Meguid *et al*. (2008) found poorer survival in right-sided cancer while the later Weiss *et al*. (2011) study found no difference [6, 19]. The latest re-analysis by Warschkow *et al*. (2016) highlighted the lack of important confounders such as MSI as a key weakness of the SEER data, a point they attempted to address with a propensity score matching methodology [11]. In the study by Warschkow *et al*. (2016), the result again was a different result, with right-sided cancer having a better prognosis. Together the prior literature suggests limitations that require a better understanding of underlying tumor biology.

The current study aimed to overcome the limitations of prior studies by performing subgroup analyses that addressed some of these shortcomings. The first subgroup analysis addressed the possibility that prognostic differences seen in individual stages from prior literature might be a result of key factors such as mismatch repair data. In several previous large studies the prognostic significance of tumor sidedness differed depending on tumor stage [6, 11]. Warschkow *et al*. (2016) for example found better prognosis in right-sided cancer in a combined stage I-III cohort but no difference in stage III cancer. In the current cohort, the only individual stage to demonstrate prognostic differences based on sidedness was stage III. We therefore analyzed stage III patients specifically in a subgroup analysis. In this analysis we found that there was a survival difference in the univariate analysis favoring left sided cancer. We then performed a multivariate analysis that included MMR status and BRAF V600E mutation status. This analysis was performed as microsatellite instability is a well-known positive prognostic marker of CRC and is known to be associated with right-sided colorectal cancer [20]. With this included in the multivariate analysis, sidedness was not found to be significant. A second sub-analysis explored this hypothesis further by re-examining the combined stage I-III cohort but with patients possessing known mismatch repair deficiency removed. When compared to the initial primary analysis, sidedness became significant in the subgroup univariate analysis but not in the multivariate analysis. These two subgroup analyses together suggest that sidedness is a surrogate for other key prognostic variables such as mismatch repair (MMR) which have often not been assessed in prior studies.

The third sub-group analysis extended on the idea that sidedness is a surrogate for key clinicopathological data by incorporating further clinicopathological data. This sub-group analysis utilized a smaller cohort of 779 patients in which additional clinicopathological variables with known independent prognostic association were included. These variables, such as the lymphocyte-to-monocyte ratio (LMR) and apical node status, have previously been investigated and shown to be independent prognostic factors in CRC [16, 21]. Most of these markers have not been previously included in studies investigating sidedness as a prognostic marker, likely due to lack of available data. In this subgroup analysis we again confirmed that tumor sidedness was not an independent prognostic marker. These findings are important as this is one of the largest cohorts to examine mismatch repair and BRAFV600E mutation status in the context of tumor sidedness. Moreover, it is the largest cohort to address sidedness and markers of the systemic inflammatory response such as the lymphocyte-to-monocyte ratio.

The secondary goal of our study was to explore tumor biological factors, their association with sidedness and whether they could explain prior findings of survival differences based on sidedness. The underlying hypothesis of the current study was that prior studies were flawed due to non-inclusion of important confounding factors. Evidence already exists for this hypothesis within the established literature. The seminal paper examining the four consensus molecular subtypes (CMS) of colorectal cancer demonstrated that sidedness was associated with differential enrichment of particular molecular subtypes [10]. In that study by Guinney *et al*. (2015), right-sided cancer was found to be more frequently than left-sided cancer to be MSI-high, CIMP-high and BRAF mutant. In our study, we re-affirmed that MMR deficiency and BRAFV600E mutation are associated with right-sided colorectal cancer, both of which have previously been shown to have prognostic value.

The current study also discovered some novel associations with sidedness that were centered on the host immune response. The first novel finding in this part of the study was that right-sided cancer was more likely to have the presence of peri-tumoral lymphocytic response (PTLR). PTLR is a reflection of the activation of the hosts’ lymphatic system against tumors and can be quantified by lymphoid aggregates at the periphery of the tumor [22]. PTLR has been associated with better prognosis and also molecular alterations such as microsatellite instability [23]. However, the composition of these lymphoid aggregates and their exact role in anti-tumor activity remains poorly defined. The second novel finding of the current study was that we demonstrated for the first time in a single study that both a low LMR and high NLR were specifically associated with right-sided CRC. These biomarkers are markers of systemic inflammation and have been well established to be independent negative prognostic markers in CRC [16, 24]. Thus, the current study suggests that right-sided cancer is associated with an elevated inflammatory state which itself is associated with poorer prognosis. These findings support the idea of cancer related inflammation as a key hallmark and driver of cancer. We can only speculate as to why right-sided cancer is associated with an elevated systemic inflammatory response. It may be that differences in the expression of certain genetic pathways predispose right-sided cancer to be more pro-inflammatory or perhaps it is the composition of gut flora in different parts of the large bowel that is responsible for a pro-inflammatory state. Regardless, this second part of the current study strongly suggests that there are competing components of the host immune response in CRC associated with survival. These factors play an integral part of the debate regarding tumor sidedness and supports an alternate hypothesis that tumor sidedness is simply a surrogate marker of other key clinicopathologic factors.

Contextually, the prognostic implications of tumor sidedness have gained prominence because of its potential to change treatment paradigms. A recent meta-analysis by Arnold *et al*. (2017) examined 6 prior studies of metastatic CRC patients treated with chemotherapy and EGFR-directed antibodies. They found that right-sidedness was not only a negative prognostic factor but also predictive of poor response to treatment [8]. These findings by Arnold *et al*. (2017) are limited to the metastatic setting and whether they are applicable to non-metastatic disease is yet to be demonstrated. However, the current study has highlighted that a better understanding of underlying tumor biology is needed before sidedness can be utilized to guide clinical management.

Compared to prior cohorts, the current cohort did have some similarities. The 5-year OS rate of 64.5% when pooled together was similar to those on large databases such as SEER [2, 25]. Stage by stage, the survival percentages were also similar. However, our cohort consisted of patients from a region of primarily high socio-economic background, a factor often associated with increased OS [26]. Patients were also more likely to be male and older than 70. The cohort spanned a long period from 1998 to 2012 including many patients treated prior to the introduction of treatments such as oxaliplatin. These differences may have attributed in part to the different results observed. One important consideration in the current study was the addition of rectal patients in the left sided cancer group. Although rectal cancer could be considered a different entity with differing treatment and natural history, we included them for several reasons. The principal reason was that the current best evidence for sidedness being a prognostic factor is the prior meta-analysis by Petrelli *et al*. (2017). This meta-analysis has also included cohorts with rectal patients [4]. Furthermore, other key studies such as the one investigating the consensus molecular subtypes and tumor sidedness have also included rectal cancer [27]. We therefore believed that incorporating rectal patients was important to make direct comparisons with prior literature. To alleviate potential concerns regarding inclusion, a sensitivity analysis was performed in which rectal patients were removed from the primary analysis and repeated. The overall result that sidedness was not an independent prognostic marker remained the same.

The study had some significant limitations that may have contributed to differences in results. Firstly, we lacked chemotherapy data on patients due to the fragmented nature of patient record keeping across multiple sites. Secondly, we had incomplete data for all clinicopathological variables. However, we chose to present all data without truncation as we believed that this would contribute more information to the current literature. Thirdly, there are inherent limitations to retrospective analysis compared to prospectively validated studies. Lastly, we also lacked data on disease free survival and cancer specific survival. Despite these limitations the study did confirm the importance of previously unaccounted tumor biological factors. Future studies in this area should focus on incorporating all key confounders such as RAS mutation status to ascertain all other clinicopathological variables for which sidedness could be a surrogate. Future studies should also consider investigating these confounders in the context of the continuum model of colorectal cancer, a model that considers change in tumor biology as gradual throughout the colon and not restricted to certain regions [28]. Recent literature has already suggested that there are important regional differences in mutation profiles and CMS beyond a left-right CRC model [29].

In conclusion, the current study has expanded the debate regarding sidedness and CRC. We found no differences in survival between left and right-sided CRC which we hypothesized was a result of previously unaccounted confounders. Significant work is still required to bring greater clarity to the question of sidedness and prognosis.
